# Emotional Reasoning Processes and Dysphoric Mood: Cross-Sectional and Prospective Relationships

**DOI:** 10.1371/journal.pone.0067359

**Published:** 2013-06-24

**Authors:** David Berle, Michelle L. Moulds

**Affiliations:** School of Psychology, The University of New South Wales, Sydney, New South Wales, Australia; University of Adelaide, Australia

## Abstract

Emotional reasoning refers to the use of subjective emotions, rather than objective evidence, to form conclusions about oneself and the world [1]. Emotional reasoning appears to characterise anxiety disorders. We aimed to determine whether elevated levels of emotional reasoning also characterise dysphoria. In Study 1, low dysphoric (BDI-II≤4; *n* = 28) and high dysphoric (BDI-II ≥14; *n* = 42) university students were administered an emotional reasoning task relevant for dysphoria. In Study 2, a larger university sample were administered the same task, with additional self-referent ratings, and were followed up 8 weeks later. In Study 1, both the low and high dysphoric participants demonstrated emotional reasoning and there were no significant differences in scores on the emotional reasoning task between the low and high dysphoric groups. In Study 2, self-referent emotional reasoning interpretations showed small-sized positive correlations with depression symptoms. Emotional reasoning tendencies were stable across an 8-week interval although not predictive of subsequent depressive symptoms. Further, anxiety symptoms were independently associated with emotional reasoning and emotional reasoning was not associated with anxiety sensitivity, alexithymia, or deductive reasoning tendencies. The implications of these findings are discussed, including the possibility that while all individuals may engage in emotional reasoning, self-referent emotional reasoning may be associated with increased levels of depressive symptoms.

## Introduction

Prevailing cognitive-behavioural models of mental disorders emphasise the influence of cognitions (automatic thoughts, beliefs and interpretations) on emotions. These models only give passing regard to the possibility that the relationship between cognitions and emotions may be bidirectional or that emotional states may influence cognitive content and processes. Beck and Emery [2] encouraged therapists to discuss with clients how they may be basing their interpretations on feelings rather than facts (suggesting an influence of feelings and emotion on cognition) and “mistaking feelings for facts” has become a standard inclusion in the “unhelpful thinking habits” sections of many cognitive-behavioural therapy (CBT) manuals [3]. However, there is little empirical research on such processes in clinical disorders.

Whether the emotional and mood states experienced in clinical disorders influence judgements and interpretations remains an open question. Arntz et al. [1] investigated whether emotional reasoning characterises anxiety disorders. Emotional reasoning is defined as a process whereby subjective emotions, rather than objective evidence, are used to determine the conclusions that an individual makes about the external world [4]. In this respect, emotional reasoning refers to more than a negative appraisal of oneself or a situation: it instead implies a process of thinking whereby emotional states are given disproportionate influence in the formation of an interpretation.

Arntz et al. [1] administered an emotional reasoning task to four different anxiety disorder groups (panic disorder, spider phobia, social phobia, and a mixed anxiety disorders group) and to a non-clinical control group. The emotional reasoning task involved participants providing ratings of the dangerousness of scenarios that varied according to whether an anxious response was or was not indicated.

Arntz et al. [1] found that, in contrast to the control group, each of the anxiety disorder groups engaged in emotional reasoning. Most interestingly, the tendency to engage in emotional reasoning was not restricted to disorder-relevant situations. These findings have been replicated in a sample of patients with posttraumatic stress disorder (PTSD) [5]. Furthermore, emotional reasoning has been associated with levels of anxiety and depression in a non-clinical child sample [6].

Emotional reasoning has similarities with the concept of anxiety sensitivity, which refers to beliefs that anxiety experiences have negative implications [7], and with alexithymia [8]. Arntz and colleagues [1] have suggested that anxiety sensitivity might lead individuals to infer danger when they experience anxiety, that emotional reasoning might cause people to fear anxiety symptoms (i.e., anxiety sensitivity), or that both anxiety sensitivity and emotional reasoning may be caused by a third factor. In addition, the relationship between emotional reasoning and the construct of alexithymia has not been investigated. Perhaps alexithymic individuals, who lack a sophisticated understanding of the sources of emotional experience, may be prone to engaging in emotional reasoning [8].

Beyond anxiety, whether or not emotional reasoning tendencies are associated with depressive symptoms in adults is yet to be determined. To the extent that clinical levels of anxiety and depression are characterised by a shared factor of negative affectivity [9] and frequently co-occur [10], it seems plausible that individuals with depression, as well as those with anxiety, might engage in emotional reasoning to a greater extent than those without. Moreover, findings that ruminating in particular ways about sad mood can maintain symptoms of depression [11], and that negative emotions in the context of life stressors might serve to reactivate depressogenic thinking in previously depressed individuals [12], suggest that emotional reasoning may also be characteristic of depression.

In anxiety, the most obvious appraisal that might be influenced by emotional reasoning is the perceived danger of a situation. Depression however, lacks a single defining appraisal or interpretative style that defines the disorder. For instance, a depressed person may reason: “If I’ve failed, I feel sad, so, if I feel sad, I must have failed”, but equally that: “If the situation is hopeless, I feel despondent, so, if I feel despondent, the situation must be hopeless”, or any number of other possibilities.

With this in mind, we hypothesised that high dysphoric individuals would engage in emotional reasoning to a greater extent than would low dysphoric individuals. We expected that emotional reasoning in dysphoric individuals might be characterised by any of a number of interpretations, but especially those pertaining to how worthless and incompetent one is, as well as how hopeless the situation is. An additional goal of Study 1 was to determine the independence of emotional reasoning from anxiety sensitivity and alexithymia, respectively.

## Materials and Methods

### Participants

Seventy first year psychology students (mean age = 19.09; *SD = *2.14; 42 [60%] female), who received course credit for their participation were divided into low dysphoric (BDI-II≤4) and high dysphoric (BDI-II ≥14) groups on the basis of their BDI-II scores. The lower cut-off of 4 for the BDI-II was chosen as this has been used in previous studies [13]. We opted for an upper cut-off of 14 on the basis that it corresponds to “mild depression” according to the BDI-II manual. Participants who scored in mid-range of BDI-II scores were excluded and participated in an alternative study.

### Ethics Statement

All participants were 17 years or older and provided informed written consent to participate in the study. The study was approved by the University of New South Wales Human Research Ethics Advisory Panel C (Behavioural; File no. 889).

### Self-report Measures

The following self-report measures were presented in randomised order to reduce the possibility of order effects:

The Anxiety Sensitivity Index (ASI) [7] is a widely-used 16-item self-report scale assessing the fear of anxiety-related symptoms.

The Beck Depression Inventory II (BDI-II) [14] is a widely-used 21-item self-report scale that assesses symptoms of depression.

The Dysfunctional Attitude Scale – Form A (DAS) [15] was used to assess beliefs that are considered to contribute to cognitive vulnerability to depression [16].

The 20-item Toronto Alexithymia Scale (TAS) [17] was used to evaluate difficulties in emotional awareness and understanding.

### Emotional Reasoning Task

Following completion of the self-report questionnaires, participants were administered the emotional reasoning task. The experimental task of Arntz et al. [1] was adapted for the present study. Arntz et al.’s procedure has been used in numerous studies of both children [18] and adults [5]. For the purpose of the present study, we developed seven scenarios that were designed to be relevant for the student sample. The themes of the scenarios included friendships, world events, exams, relationships and other social situations. These were presented to participants on computer. Six of these were dysphoria-relevant and one was the panic attack themed anxiety-relevant scenario as reported in the Arntz et al. [1] paper. We included this anxiety scenario used by Arntz et al. to allow a partial replication of their study and to determine whether dysphoric individuals engage in emotional reasoning when experiencing fear.

### Emotional Reasoning Task Instructions

Each participant was asked to imagine themselves in the situation described in each scenario which was presented with four separate endings: one objectively neutral and with a non-valanced emotional response, one objectively neutral with a dysphoric (or anxious) emotional response, one with an objectively negative ending and non-valenced emotional response, and one with an objectively negative ending with a dysphoric (or anxious) emotional response (See Text S1, for an example).

### Item Validation

To ensure that our scenarios were relevant to depression and dysphoria, we surveyed psychologists (*n* = 8), clinical psychologists (*n* = 14) and psychiatrists (*n* = 2) who had been practising for an average of 9.29 years (*SD* = 7.96, range 0 to 26 years). Respondents were asked to rate the valence (from negative [−5] to neutral [0] to positive [5] on an 11 point rating scale) of each emotion-based statement from each item (e.g., “You feel sad”). They were then asked to provide a rating of how characteristic they believed that each statement was of a person who has a clinical depression (i.e., the sort of thought or feeling that a depressed person would be likely to report during a clinical assessment; 0 =  “Not at all characteristic” to 10 =  “Extremely characteristic”). When each of the means for the valence ratings were put in rank order, each of the neutral valence script-ending statements had a more positive valence rating than each of the negative script-ending statements. Likewise, for the ratings of how characteristic each statement was of clinical depression, each of the negative script statements were rated as more characteristic of depression than each of the neutral scripts.

### Emotional Reasoning Task Ratings

Following the administration of each script, participants were asked to provide the following ratings for each scenario on a 0 to 100 visual analogue scale:

1. How unfortunate is this situation?
2. How negative is this situation?
3. How worthless does this situation suggest that you are?
4. How incompetent does this situation suggest that you are?
5. How hopeless is the situation?
6. How controllable is the situation?

Mean scores for the two self-referent ratings (i.e., how worthless and incompetent the situation suggests that one is), were based on only three of the scenarios that were considered relevant for these ratings.

However, for the anxiety relevant scenario, the same ratings as provided in the Arntz et al. [1] study were used (how dangerous is the situation, how safe is the situation, how controllable is the situation, how unpleasant is the situation, and how good is this outcome). Arntz et al. reported that aside from the perceived dangerousness ratings, their other four ratings were included only as “filler” items (p. 920). We therefore limit our discussion to perceived dangerousness so far as the anxiety-related item is concerned, so as to allow comparison with the findings of Arntz et al.

Participants were presented with each of the scenario endings in one of eight pseudo-random orders such that no two endings from the sample scenario were presented consecutively (to reduce any carry-over effects).

### Data Analysis

All analyses were conducted using the Statistical Package for Social Sciences (SPSS 17.0). We compared demographic and self-report questionnaire scores using *t*-tests (for continuous variables) and chi-square tests (for categorical variables). We used the False Discovery Rate method [19] to control the Type 1 error rate in instances where multiple comparisons were made.

We calculated emotional reasoning (difference) scores as outlined by Engelhard et al. [5]. This involved subtracting the mean ratings of each scenario that involved an emotionally neutral response from the mean ratings of each situation that included an emotionally valenced response. We averaged these difference scores across the objectively neutral and objectively negative script endings to ensure that emotional reasoning scores were not confounded by the objective nature of the situation.

## Results

The high dysphoric group comprised 42 participants and the low dysphoric group 28 participants. Table 1 summarises the demographic characteristics and self-report scores for the sample.

**Table 1:** Demographic variables and self-report questionnaire scores for the low dysphoric, high dysphoric and total sample in Study 1.

		Low dysphoric (*n* = 28)		High dysphoric (*n* = 42)		Total sample (*N* = 70)		Comparing proportion of females in Low vs High dysphoric groups	
		*n*	%	*n*	%	*N*	%	?^2^	*p*-value
Females		14	50.00	28	66.67	42	60.00	1.94	0.16
								**95% Confidence Interval for Low vs High dysphoric groups**	
		**Mean**	***SD***	**Mean**	***SD***	**Mean**	***SD***	***t***	***p*** **-value**
Age		18.82	1.22	19.26	2.58	19.09	2.14	0.84	0.40
Self-report questionnaires:									
ASI	16.29		9.79	27.02	11.64	22.73	12.09	4.02	<0.0001
DAS (A)	104.82		20.50	140.81	32.52	126.41	33.29	5.20	<0.0001
TAS – Total score	41.07		8.58	55.26	11.38	49.59	12.44	−5.61	<0.0001

ASI = Anxiety Sensitivity Index; DAS (A) = Dysfunctional Attitudes Scale – Form A; TAS = Toronto Alexithymia Scale.

There were no significant differences in emotional reasoning scores between each of the eight different orders of presentation of the emotional reasoning task (all *p*s >.05), suggesting that there were no order of presentation effects. The emotional reasoning scores for each rating within each of the low and high dysphoric groups were in almost all cases significantly greater than zero, suggesting that individuals within both the high and low dysphoric groups engaged in emotional reasoning (the means and standard deviations for each item of the emotional reasoning task are available from the authors on request). The exception was for ratings of controllability which were not significantly greater than zero for either the high or low dysphoric groups.

**Figure 1:**
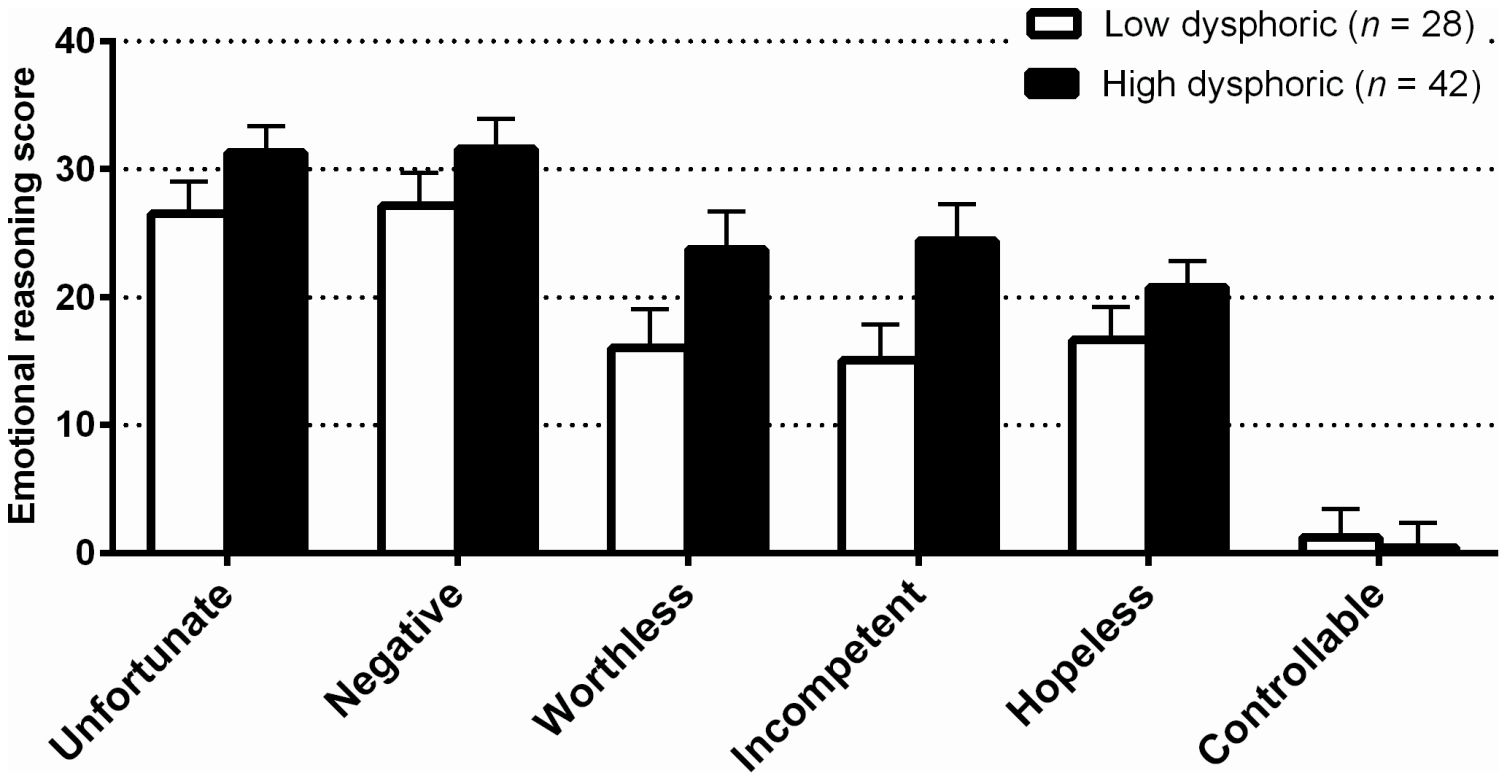
Emotional reasoning scores for high and low dysphoric groups in Study 1. Error bars are the standard error of the mean. No between group differences remained significant at *p*<0.05 after applying the False Discovery Rate for multiple comparisons.

Figure 1 shows the emotional reasoning scores in the high and low dysphoria groups. Between-group differences in mean emotional reasoning scores did not reach statistical significance after applying the False Discovery Rate for multiple comparisons. Ratings of how incompetent one was in each situation approached significance (*t* = 2.27, *df* = 1, 68, *p* = .03; ns after applying the False Discovery Rate). We repeated these analyses after excluding one of the scenarios that referred to feeling “worthless and hopeless” as these were also ratings that participants provided for each of the situations. When we did this, the pattern of results was the same.

So far as the anxiety-related rating was concerned, the between-group difference in mean emotional reasoning scores was not significant.

There were no significant correlations between emotional reasoning and scores on any of the self-report measure scores indicating that emotional reasoning scores were independent of dysfunctional attitudes, anxiety sensitivity, and alexithymic tendencies (see Table 2).

**Table 2:** Correlations between emotional reasoning scores^a^ for the dysphoria items and self-report measures in Study 1.

Emotional reasoning rating	DAS (A)	ASI	TAS – Total score
Low dysphoric (*n* = 28)			
Unfortunate	0.03	−0.08	0.13
Negative	0.17	−0.08	0.13
Worthless	0.36	−0.03	0.03
Incompetent	0.30	−0.15	0.08
Hopeless	0.03	−0.10	0.22
Controllable	0.20	0.50	0.17
High dysphoric (*n* = 42)			
Unfortunate	−0.32	−0.19	−0.22
Negative	−0.15	−0.18	−0.27
Worthless	−0.07	−0.07	−0.13
Incompetent	−0.01	−0.03	−0.09
Hopeless	−0.30	−0.23	−0.24
Controllable	0.30	0.15	0.20

No correlations were significant after applying the False Discovery Rate.

DAS (A) = Dysfunctional Attitudes Scale – Form A; ASI = Anxiety Sensitivity Index; TAS = Toronto Alexithymia Scale.

a Emotional reasoning scores were calculated by subtracting ratings for situations without a negative emotional response from ratings for situations with a negative emotional response.

## Discussion

The findings of this study indicated that both the low and high dysphoric groups engaged in emotional reasoning, as evidenced by more negative ratings for the scenarios that indicated a negative emotional response. However, with few exceptions, scores on the emotional reasoning task were not significantly greater for the high dysphoric group than the low dysphoric group. It is noteworthy that one of the self-referent ratings - incompetence - approached significance (*p*>.05 after controlling for multiple comparisons).

It is also noteworthy that there was no significant difference between the high and low dysphoric groups for the dangerousness rating for the anxiety-themed script. Although Arntz et al. [1] found that emotional reasoning was a transdiagnostic tendency, in that each of their different anxiety-disorder groups demonstrated emotional reasoning, our findings suggest that dysphoric individuals do not engage in emotional reasoning in anxiety-themed situations. Admittedly, our high dysphoric group may have been below the threshold for a clinical disorder, and this might account for our non-significant findings. We also administered only one of Arntz et al.’s four emotional reasoning items, so a more extensive replication of their findings is needed.

Emotional reasoning scores were also independent of scores on the measures of dysfunctional attitudes, anxiety sensitivity and alexithymic tendencies. To the extent that the scenarios procedure used in our study was a valid measure of emotional reasoning, the absence of these associations in our data indicates that emotional reasoning may be a separate construct and that our findings may not have been confounded by participants with high levels of each these tendencies.

These findings could be extended in several ways. First, the inclusion of additional self-referent ratings for each scenario would allow us to be more confident that there is indeed a tendency for self-referent ratings, as opposed to other ratings, to be especially influenced by mood in high dysphoric participants. Second, the procedure used in this initial study did not allow us to determine whether emotional reasoning tendencies might simply be accounted for by more general deficits in deductive reasoning, rather than a style of reasoning that is specific to emotional states. Third, the predictive value of emotional reasoning across time could not be ascertained from our results. In this regard, emotional reasoning tendencies might have value for predicting subsequent depressive symptoms. We aimed to address each of these limitations in our second study.

### Study 2

Recent conceptualisations of cognitive processes in depression have placed an emphasis on negative self-referent interpretations as particularly characteristic of the disorder. For instance, negative views of the world and the future in depression may be limited to one’s own self and future [20], helplessness theories of depression highlight internal attributions and self-blame in depression [21], the response-styles theory focuses on internal self-analysis of the causes of depression [22], and diagnostic criteria place importance on one’s feelings of worthlessness (DSM-IV) [23].

Given the apparent importance of such self-referent interpretations in depression, we therefore decided to include relatively more self-referent ratings in this second study (i.e., ratings of how pathetic and inadequate participants would consider themselves to be in the given situation, as well as ratings of perceived worthlessness and incompetence). For the purpose of replication of our results from Study 1, we retained two non-self referent ratings: the degree to which participants perceived the situation as unfortunate and the degree to which the situation was interpreted as negative.

We also extended the previous study by including a deductive reasoning task and by re-assessing participants 8 weeks later. By including the reasoning task, we hoped to confirm that emotional reasoning tendencies are independent of deductive reasoning ability. In addition, the re-assessment of participants 8 weeks later allowed us to determine whether emotional reasoning predicts subsequent depressive symptoms. Prospective relationships have not been previously investigated in an adult sample. A previous study of children and adolescents however, found that emotional reasoning predicted subsequent anxiety symptoms [24].

Eligibility for Study 1 had been determined on the basis of BDI-II scores. However, for this second study we recruited participants regardless of BDI-II score. Thus, the sample comprised the full spectrum of BDI-II scores. This allowed us to 1). conduct analyses using BDI-II scores as a continuous variable and 2). replicate the analyses of Study 1 by dividing the sample into high and low dysphoric groups (BDI-II≤4 [low dysphoric]; BDI-II ≥14 [high dysphoric]).

Further, in Study 2 we assessed participants’ levels of anxiety symptoms. Given the frequent co-occurrence of anxiety and depressive symptoms, we measured anxiety in order to control for these symptoms and thus rule out the possibility that any differences in emotional reasoning that emerged between high and low dysphoric individuals were merely a consequence of anxiety symptoms (i.e., given the findings of Arntz et al. [1]).

For Study 2 then, we aimed to determine whether depressive symptom scores are correlated with emotional reasoning tendencies. In this respect, we hypothesised that there would be significant positive correlations between BDI-II scores and emotional reasoning scores, particularly for the self-referent emotional reasoning ratings (i.e., worthless, incompetent, pathetic and inadequate) that we expected might be especially characteristic of depression.

We also aimed to replicate and extend the findings of Study 1. Our second hypothesis was that, consistent with the findings of Study 1, emotional reasoning scores would be significantly greater than zero. We also hypothesised that, with a larger sample and additional self-referent ratings, the high dysphoric group would score significantly higher on the emotional reasoning task than the low dysphoric group for the self-referent emotional reasoning ratings. In an extension of Study 1, we also assessed current anxiety symptoms to allow greater confidence that any differences in emotional reasoning scores between high and low dysphoric participants were not an artefact of differences associated with anxiety symptoms. Our fourth hypothesis was that the high anxiety symptoms group would score significantly greater on the emotional reasoning task than the low anxiety symptoms group, consistent with Arntz et al.’s [1] findings that clinical levels of anxiety are associated with greater levels of emotional reasoning, regardless of the context in which emotional reasoning may arise (e.g., in panic-related, social anxiety-related, or in this case, dysphoria-related situations). Fifth, we hypothesised that emotional reasoning scores would be independent of deductive reasoning abilities. Sixth, we hypothesised that scores on the emotional reasoning task would be stable across an 8 week interval, consistent with previous notions that emotional reasoning may be a trait-like tendency [1]. Finally, given that emotional reasoning tendencies have been associated with subsequent anxiety symptoms in children and adolescents, we hypothesised that scores on the emotional reasoning task would predict depressive symptoms 8 weeks later.

## Methods

### Participants

Participants were 118 first year psychology students (91 females; 77.1 percent; mean age = 19.79, *SD* = 4.94) who participated in exchange for course credit.

The mean interval from baseline to the second session was 7.80 weeks (Median = 8.00, *SD* = 0.93). The follow-up interval of 8 weeks was chosen for practical reasons in that participants could be re-assessed within the same semester of their course so as to reduce the rate of attrition. Of the 118 participants who completed the baseline assessment, 106 (89.8%) also completed the second session. Data regarding the reasons for not participating in the second session were not systematically collected, although it was noted that a number of those who did not wish to attend the second session had already earned their necessary research participation credit for the semester and so were not interested in attending the second session. Importantly, participants who did and did not complete the second session did not differ statistically in demographic characteristics or self-report scores. Participants who did not attend the follow-up scored more highly on baseline emotional reasoning pertaining to how “pathetic” situations suggested that they were (*t* = 2.30, *df* = 116, *p* = .02) but were otherwise equivalent on other baseline emotional reasoning ratings.

### Ethics Statement

All participants were 17 years or older and provided written informed consent to participate in the study. The study was approved by the University of New South Wales Human Research Ethics Advisory Committee Panel C (Behavioural; File no. 1056).

### Self-report Measures

The Beck Anxiety Inventory (BAI), BDI-II and DAS were administered at the baseline assessment and the BAI and BDI-II were administered at the second assessment.

The BDI-II and DAS are described in Study 1. The BAI [25] is a widely used 21-item self-report scale that assesses common features of anxiety, such as nervousness, a fear of losing control, and somatic aspects of anxiety.

### Procedure

For Study 2 we decided to replace four scenarios that did not seem likely to give rise to self-referent emotional reasoning with four additional new ones that did (available from the authors on request). We repeated the same item validation process as in Study 1 and this again confirmed the valence and relevance of each item for depression. Participants were asked to provide ratings for how unfortunate and negative each respective situation was, as well as how worthless, incompetent, pathetic and inadequate each situation suggested that they were, with the latter four ratings considered to be “self-referent”.

For the anxiety related scenario, the dangerousness rating was again used.

To ensure that emotional reasoning tendencies are not simply an artefact of a more general deductive reasoning deficit, we also administered a modified version of the Wason Selection Task (WST) [26]. The task was modified so that the hypothetical situations had local and age appropriate relevance (e.g., establishing whether one hour of study each night will necessarily lead one to receive a distinction in one’s psychology course). Each participant was instructed to select the minimum number of (four) “cards” (on a computer screen) to “turn over” to disconfirm the stated rule, with each card indicating either whether the antecedent had occurred (or not) or whether the consequence had occurred (or not). For instance, for the study related item, the correct response would be to “turn over” the cards “Did study for an hour each night” and “Did not receive a distinction”. The task generates scores for verification and falsification, as well as a total score (higher scores indicating better deductive reasoning ability).

### Follow-up

At the second session, the above procedure was repeated except that the WST was not readministered.

### Data Analysis

To allow replication of the findings from Study 1, we divided the sample into high (BDI-II ≥14; *n* = 34) and low (BDI-II≤4; *n* = 20) dysphoric groups based on the BDI-II. Although not a focus of the present study, we also divided the sample into high (BAI ≥16) and low (BAI≤7) anxiety groups, based on their scores on the BAI [27]. These thresholds were chosen because the manual of the BAI [27] describes scores of 7 or less as indicative of a “minimal” level of anxiety and scores of 16 and above as reflecting a “moderate to severe” level of anxiety.

As in Study 1, we calculated emotional reasoning difference scores in a similar manner to Engelhard et al. [5]. Emotional reasoning scores were then correlated with scores on other self-report measures at the initial and second assessments.

Given that the sample was not restricted according to BDI-II scores, as was the case in Study 1, we also conducted correlation analyses between emotional reasoning scores and other relevant variables (for instance, BDI-II and BAI). So far as the predictive value of emotional reasoning scores was concerned, we also correlated emotional reasoning scores at baseline with emotional reasoning scores at follow-up, and conducted partial correlations between emotional reasoning scores at baseline and BDI-II scores at follow-up, controlling for baseline BDI-II scores.

## Results

There were no differences in emotional reasoning scores according to order of presentation, except for the anxiety related scenario of perceived dangerousness (*F* = 3.39, *df* = 7, 98, *p* = .003).

### Baseline

The correlations between the self-report measures (BAI, BDI-II, and DAS) and emotional reasoning scores at baseline and follow-up are reported in the upper and lower sections of Table 3, respectively. At baseline, three of the four self-referent emotional reasoning ratings (i.e., incompetent, pathetic, and inadequate) were positively and significantly associated with BDI-II scores, although the associations were typically only small to medium in size in most cases. Thus, the first hypothesis was partially supported. Each of the self-referent emotional reasoning scores was also negatively correlated with DAS scores, suggesting that greater levels of self-referent emotional reasoning were associated with more dysfunctional attitudes.

**Table 3:** Correlations between emotional reasoning scores^a^ and BDI-II, BAI and DAS scores at the baseline and follow-up assessments in Study 2.

Baseline (*n* = 118)				
Emotional reasoning score:	BDI-II	BAI	DAS^b^	Mean (*SD*)
Unfortunate	.16	.07	−.01	30.81 (14.60)
Negative	.12	−.02	.04	34.12 (16.41)
Worthless	.18	.11	−.25*	19.41 (16.41)
Incompetent	.23*	.17	−.23*	18.35 (15.20)
Pathetic	.26*	.16	−.31*	16.84 (15.14)
Inadequate	.33*	.21	−.33*	16.84 (13.55)
Emotional reasoning anxiety score:				
Dangerous	−.06	.01	.03	9.80 (23.13)
Mean (*SD*)	11.69 (8.18)	12.42 (8.65)	194.34 (28.79)	
**Follow-up (** ***n*** ** = 106)**				
Emotional reasoning score:	BDI-II	BAI		
Unfortunate	0.06	−0.04		30.46 (16.73)
Negative	0.13	−0.03		32.28 (16.80)
Worthless	0.15	0.06		21.34 (18.86)
Incompetent	0.20	0.09		20.96 (17.63)
Pathetic	0.24	0.08		18.20 (17.19)
Inadequate	0.18	0.02		18.22 (15.91)
Emotional reasoning anxiety score:				
Dangerous	0.03	0.09		13.06 (24.11)
Mean (*SD*)	11.25 (8.59)	9.86 (9.44)		

BAI = Beck Anxiety Inventory; BDI-II = Beck Depression Inventory – II; DAS = Dysfunctional Attitudes Scale Form A.

* Significant at p<0.05 after applying the False Discovery Rate.

a Emotional reasoning scores were calculated by subtracting ratings for situations without a negative emotional response from ratings for situations with a negative emotional response.

b The dysfunctional attitudes scale – form A (DAS-A) was not administered at the follow-up assessment. Negative correlations indicate a positive association between dysfunctional attitudes and emotional reasoning scores.

**Figure 2:**
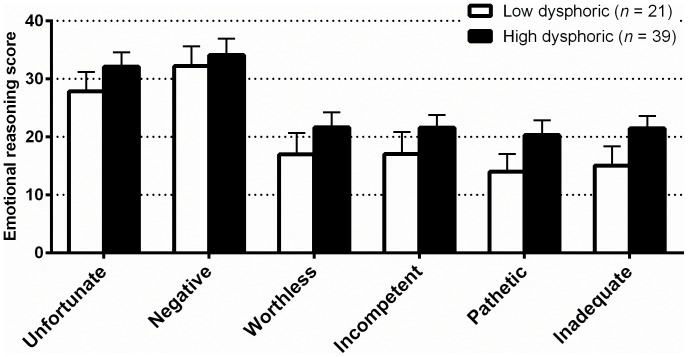
Emotional reasoning scores for the high and low dysphoric groups at baseline in Study 2. Error bars are the standard error of the mean. No between group differences were significant at *p*<0.05.

We then divided participants into high and low dysphoric groups based on their BDI-II scores at the first session (henceforth referred to as “baseline”). Consistent with our second hypothesis, participants in both groups appeared to engage in emotional reasoning (in that their emotional reasoning scores were in each case significantly greater than zero, the exception being for ratings of safety in the high dysphoric group). The means of each of the depression-related emotional reasoning scores for the high dysphoric group were greater than for the low dysphoric group, although these differences did not reach statistical significance (see Figure 2). For the anxiety-related emotional reasoning items, the low dysphoric group scored higher than the high dysphoric group for the dangerousness rating.

**Figure 3:**
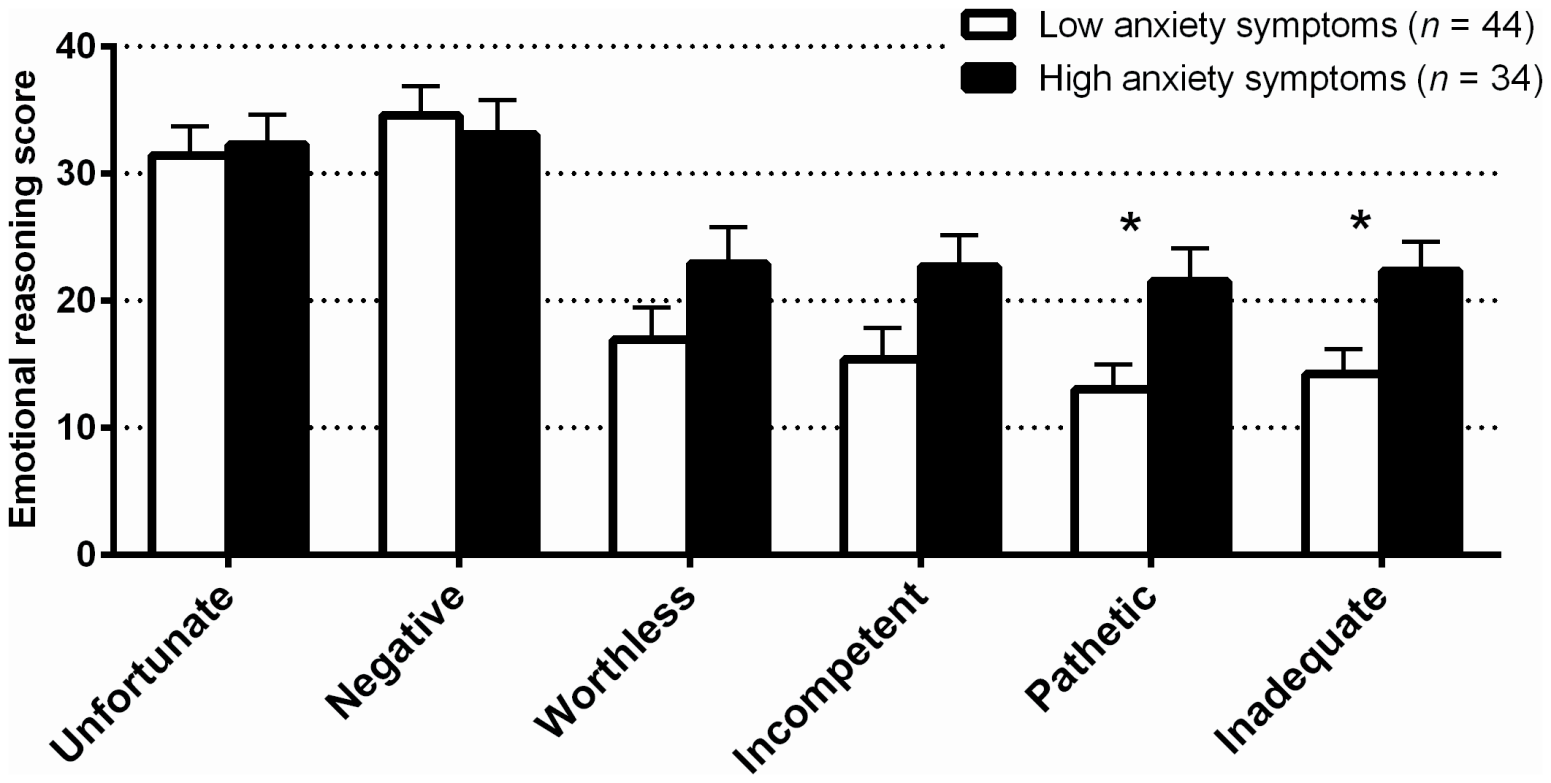
Emotional reasoning scores for the high and low anxiety symptoms groups at baseline in Study 2. Error bars are the standard error of the mean. *Between group differences were significant at *p*<0.05 after controlling for multiple comparisons.

The mean of each of the depression-related emotional reasoning scenarios was significantly greater for the high anxiety symptoms group than for the low anxiety symptoms group for ratings of “pathetic” and “inadequate” (partially supporting our fourth hypothesis; see Figure 3). There were no differences between the low and high anxiety symptoms groups for the anxiety-related scenario.

There was overlap in the composition of the high dysphoric and high anxiety symptoms groups, such that high BDI-II scorers also tended to score high on the BAI, and low BDI-II scorers tended to also score low on the BAI (χ^2^ = 24.07, *df = *1, *p*<0.001). To ensure that emotional reasoning differences between the high and low dysphoric groups were not a result of anxiety rather than depressive symptoms, we therefore conducted post-hoc partial correlation analyses to determine whether the association between BDI-II and emotional reasoning scores remained after controlling for BAI scores. None of these partial correlations were greater than ±0.10 or were statistically significant.

So far as the WST is concerned, all correlations between emotional reasoning scores and each of the WST scores (for verification, falsification and WST total score) at baseline were less than.25 and only two were significant. These were for the association between WST falsification scores and emotional reasoning scores for pathetic (*r* = .20, *p*<.05) and inadequate (*r* = .24, *p*<.01), respectively. Thus, our fifth hypothesis was not supported.

### Follow-up

Each of the emotional reasoning scores at baseline was significantly correlated with their respective score at follow-up (*r*s ranging .53 to .74). The correlations between baseline and follow-up emotional reasoning scores for the perceived dangerousness of the anxiety scenario was .21 which was not significant after applying the False Discovery Rate.

Partial correlations between (i) emotional reasoning scores at the initial assessment and (ii) BDI-II and BAI scores at follow-up, controlling for the baseline BDI-II and BAI score respectively, were each small in magnitude and none of them reached statistical significance.

## General Discussion

The findings of these two studies suggest that most individuals, even those who do not meet criteria for a mental disorder, engage in emotional reasoning. This is consistent with our first hypothesis in Study 2. In this respect, our findings are discrepant from those of Arntz et al. [1] but concordant with those reported by Engelhard et al. [5]. It is noteworthy, however, that even in the Arntz et al. study there was a trend for non-anxious participants to engage in emotional reasoning, as evidenced by non-significantly greater danger ratings when an anxious script ending was included. Together, these findings suggest that emotional reasoning may characterise all individuals to a greater or lesser extent.

The central hypothesis of each of our studies was that scores on the emotional reasoning task would be associated with greater levels of depressive symptoms. When comparing the low and high dysphoric groups in Studies 1 and 2, the direction of the between group differences was in the predicted direction in almost every case (the exception being ratings of “negative” for the high versus low anxiety symptoms comparison in Study 2), although the differences did not reach statistical significance after corrections for multiple comparisons. The high within-group variability for each of the high and low dysphoric groups may explain the lack of significant differences. It is remarkable, however, that the between group differences of the greatest magnitude were for self-referent items (i.e., incompetent and worthless in Study 1, and pathetic and inadequate in Study 2). When we conducted a more sensitive correlation analysis (i.e., which included continuous rather than categorical variables) of the Study 2 data at baseline, scores on three of the four self-referent emotional reasoning ratings were significantly correlated with depressive symptoms. At the follow-up assessment, these associations did not reach statistical significance, but were still of similar (small-sized) magnitude. Our findings therefore provide partial support for the second hypothesis of Study 2, in that scores for self-referent emotional reasoning may be especially likely to be elevated among participants with high levels of depressive symptoms.

We included the panic disorder-relevant scenario from Arntz et al. [1] in each of our studies to determine whether our samples would engage in emotional reasoning in anxiety-provoking situations as well as in dysphoria-relevant situations. In contrast to the findings of Arntz et al., the scores of our participants on this item did not suggest that they were engaging in anxiety-related emotional reasoning. This may be due to the fact that neither of our university-student samples were comprised of predominantly clinical participants unlike in the Arntz et al. study. Further replication is therefore needed, especially given that we included only the panic disorder-relevant script of Arntz et al., and not the two that were tailored for other disorders (i.e., social anxiety disorder and specific phobia), or their non-specific “control” script.

Aside from establishing whether participants with high and low levels of depressive symptoms respond differently to anxiety-related situations, our second study also investigated whether individuals with high levels of anxiety symptoms had greater dysphoria-relevant emotional reasoning scores than those with low levels of anxiety symptoms. Interestingly, two of the four self-referent emotional reasoning ratings (pathetic and inadequate) were significantly elevated in the high anxiety symptoms group, despite the thematic similarities between these items and depression or low self-esteem, rather than anxiety. This raises the possibility that some of the associations between emotional reasoning and depression could be accounted for by co-occurring anxiety symptoms. However, when we conducted a partial correlation analysis between emotional reasoning scores and depressive symptoms, controlling for anxiety symptoms, the magnitude of the correlations between emotional reasoning scores and depressive symptoms was only marginally reduced. This provides preliminary evidence to suggest that associations between emotional reasoning and depressive symptoms are not simply an artefact of the overlap between depressive and anxiety symptoms.

The results of Study 1 allow increased confidence that emotional reasoning is not simply a reflection of high levels of anxiety sensitivity. Likewise, the lack of a significant association between emotional reasoning and alexithymia scores indicate that emotional reasoning was independent of participants’ ability to recognise and understand emotions. Perhaps individuals with high levels of emotional reasoning are able to recognise and understand their emotions effectively, but place an inflated importance on the implications of such emotions. Finally, consistent with our fifth hypothesis from Study 2, emotional reasoning scores were independent of deductive reasoning ability, the small-sized associations between falsification scores and ratings of pathetic and inadequate notwithstanding. To the extent that falsification scores may correspond to an ability to disconfirm assumptions, this ability may help to protect individuals who are prone to self-referent emotional reasoning from experiencing more severe depressive symptoms if they are otherwise able to seek exceptions to a negative interpretation of their mood states. We acknowledge that this account is merely speculative at this stage.

Consistent with suggestions by other researchers that emotional reasoning might be a trait-like tendency [1], we found that scores on the emotional reasoning task were consistent across the 8-week follow-up interval (Study 2, hypothesis 5). Given the seemingly complex developmental trajectories of emotional reasoning during childhood [24], [28], the findings of the present study indicate that, at least by early adulthood, emotional reasoning tendencies might be relatively entrenched.

There were no significant correlations between emotional reasoning scores at baseline and depressive symptom scores at follow-up, controlling for baseline levels of depressive symptoms. Thus, the seventh hypothesis of Study 2 was not supported. Subsequent anxiety symptoms were also not predicted by emotional reasoning scores. This raises the possibility that emotional reasoning tendencies may not portend either the recurrence or persistence of depressive (or anxiety) symptoms. One possibility is that emotional reasoning may contribute to the initial occurrence of depressive symptoms, and that other factors, for instance, withdrawal from rewarding activities, may play a part in the persistence or recurrence of such symptoms.

One interesting possibility to consider is that it may not necessarily be the case that emotional reasoning is an unhelpful tendency in all situations. The aforementioned finding that even participants with low levels of depressive and anxiety symptoms demonstrated emotional reasoning difference scores of greater than zero suggests that emotional reasoning in and of itself may not be a sign of psychopathology. There may be situations where one’s emotional state provides important and helpful information for the situation at hand, and research by social psychologists has emphasised the potentially adaptive function of allowing one’s emotional and affective state to guide cognitive processing [29]. One possibility is that it may instead be the degree of emotional reasoning or the extent to which an individual’s emotional state information impedes the processing of other important information about the situation which puts the individual at risk for increased depressive or anxiety symptoms.

There are numerous limitations of the studies reported here. First, we relied on student samples. Replication of these results in a clinical sample would allow hypotheses about the relationship between emotional reasoning and clinical depression to be tested. Second, these two studies were only able to investigate associations, and whether there is a causal precedence of emotional reasoning or depressive symptoms over the other remains unclear. Still, our preliminary prospective findings suggest that scores on the emotional reasoning task may not necessarily predict subsequent depressive symptoms. On the other hand, it remains possible that elevated levels of anxiety or depressive symptoms put an individual at risk of increased emotional reasoning. Third, perhaps the most noteworthy limitation of the present studies was that the script-based emotional reasoning procedure that we employed, although used in numerous previous studies, has not previously been systematically validated. Thus, the construct validity of the task remains to be verified and we acknowledge that there is a lack of ecological validity in that participants were required to imagine themselves feeling certain emotions in particular situations [30]. Other studies have used ambiguous biofeedback procedures to investigate anxiety-based emotional reasoning [31], however, the lack of well validated procedures for measuring depression-relevant emotional reasoning in the published literature calls for the development of additional experimental procedures and self-report methods.

In summary, our two studies suggest that there may be small-sized associations between emotional reasoning and depressive symptoms. This association appears to be independent of anxiety symptoms. Emotional reasoning appears most pronounced when individuals make self-referent interpretations of situations. Although emotional reasoning appears to be a stable tendency, there remains uncertainty regarding the prospective value of emotional reasoning in predicting depressive symptoms. With further validation of emotional reasoning measures as well as replication of these findings using clinical samples and alternative emotional reasoning procedures, the links between emotional reasoning and depression may be better understood.

## Supporting Information

Text S1 **Example of emotional reasoning script used in Study 1.**
(DOCX)
