# Supplementary material for: Emotional Reasoning Processes and Dysphoric Mood: Cross-Sectional and Prospective Relationships
Source: PLoS One. 2013 Jun 24;8(6):e67359. doi: 10.1371/journal.pone.0067359 (PMC3691160; doi:10.1371/journal.pone.0067359)
Supplement: Text S1 — Example of emotional reasoning script used in Study 1. (DOCX) [file pone.0067359.s001.docx]

1. You receive a phone call from your best friend of many years. She tells you that she is thinking about her options in life. You feel composed. She is obviously thinking through what she wants to do with her life. (Objectively neutral situation and non-valenced emotional response).
2. You receive a phone call from your best friend of many years. She tells you that she is thinking about her options in life. You feel sad. She obviously isn’t feeling content these days. (Objectively neutral situation and negatively valenced emotional response).
3. You receive a phone call from your best friend of many years. She tells you that she is moving overseas. You feel composed as you remind yourself that she is pursuing her goals. (Objectively negative situation and non-valenced emotional response).
4. You receive a phone call from your best friend of many years. She tells you that she is moving overseas. You feel sad as you will miss her company. (Objectively negative situation and negatively valenced emotional response).
